# Combating Antibiotic Resistance through the Synergistic Effects of Mesoporous Silica-Based Hierarchical Nanocomposites

**DOI:** 10.3390/nano10030597

**Published:** 2020-03-24

**Authors:** Ranjith Kumar Kankala, Wei-Zhi Lin, Chia-Hung Lee

**Affiliations:** 1 Department of Life Science, National Dong Hwa University, Hualien 97401, Taiwan; ranjithkankala@hqu.edu.cn (R.K.K.); ex20122@gmail.com (W.-Z.L.); 2 College of Chemical Engineering, Huaqiao University; Xiamen 361021, China

**Keywords:** pH-sensitive release, copper, mesoporous silica nanoparticles, antibiotic resistance, silver nanoparticles, polyethyleneimine, tetracycline

## Abstract

The enormous influence of bacterial resistance to antibiotics has led researchers toward the development of various advanced antibacterial modalities. In this vein, nanotechnology-based devices have garnered interest owing to their excellent morphological as well as physicochemical features, resulting in augmented therapeutic efficacy. Herein, to overcome the multidrug resistance (MDR) in bacteria, we demonstrate the fabrication of a versatile design based on the copper-doped mesoporous silica nanoparticles (Cu-MSNs). Indeed, the impregnated Cu species in the siliceous frameworks of MSNs establish pH-responsive coordination interactions with the guest molecules, tetracycline (TET), which not only enhance their loading efficiency but also assist in their release in the acidic environment precisely. Subsequently, the ultrasmall silver nanoparticles-stabilized polyethyleneimine (PEI-SNP) layer is coated over Cu-MSNs. The released silver ions from the surface-deposited SNPs are capable of sensitizing the resistant strains through establishing the interactions with the biomembranes, and facilitate the generation of toxic free radicals, damaging the bacterial components. In addition to SNPs, Cu species impregnated in MSN frameworks synergistically act through the production of free radicals by participating in the Fenton-like reaction. Various physical characterization techniques for confirming the synthesis and successful surface modification of functional nanomaterials, as well as different antibacterial tests performed against MDR bacterial strains, are highly commendable. Remarkably, this versatile formulation has shown no significant toxic effects on normal mammalian fibroblast cells accounting for its high biocompatibility. Together, these biocompatible MSN-based trio-hybrids with synergistic efficacy and pH-responsive delivery of antibiotics potentially allow for efficient combat against MDR in bacteria.

## 1. Introduction

Recently, the rapid upsurge of antibiotic resistance has become a significant clinical therapeutic burden due to numerous defense mechanisms developed by microorganisms to counteract antimicrobials [1]. These consequences eventually reduce the efficacy of antibiotics, leading to the accompanying changes in the therapeutic dosage regimen owing to the poor understanding and utilization of excess amounts of drugs or different antibiotics for combinatorial therapy [2]. To overcome this catastrophe, a few potential steps can be applied, such as discovering new antibiotics or materials possessing excellent abilities for long-term treatment efficacy [2,3], and improving drug delivery with targeting ability to act against antibiotic resistance. Nevertheless, although numerous antibiotics have been available, only some of those are highly effective against various resistant bacterial strains. Moreover, the progress for the discovery of new chemical entities and their subsequent approval is time-consuming and expensive. These significant limitations motivated the researchers toward utilizing the nanotechnology-based devices for developing the antibacterial modalities due to their abundant surface chemistry and high surface-to-volume ratio, accounting for ease of surface functionalization for immobilizing targeting ligands as well as encapsulating various therapeutic guests, respectively [4,5,6]. The use of porous nanoparticles offers numerous advantages, such as safeguarding the sensitive guest molecules in the harsh microenvironment, improving the aqueous solubility of hydrophobic drugs, fabricating stimuli-responsive release, and controlled as well as targeted drug delivery approaches. Such advancements based on nanoparticles significantly enhance the availability of drugs at the infectious sites, either passively through enhanced permeation and retention effect (EPR), or actively by utilizing precise ligands [7,8]. In this vein, several nanoparticle-assisted delivery systems have been formulated for the delivery of antibiotics, such as liposomes, polymeric carriers, and various inorganic nanoparticles, among others [7,9]. However, the intrinsic instability of organic materials-based devices, such as polymers as well as liposomes, often hampered their applicability.

Amongst the diverse, inorganic-based nanoparticles available, mesoporous silica-based nanoparticles (MSNs) have gathered vast interest owing to their advantageous morphological features and attractive physicochemical properties, such as uniform and discrete hexagonal architectures, controllable sizes and shapes, tunable mesostructures, high surface area, and pore volume, as well as flexible nanospaces for surface functionalization [6,10,11,12,13]. Other characteristic features of MSNs include excellent biocompatibility and high drug-loading ability, among others. These MSNs can be used in numerous biomedical applications, such as biocatalysis, bioimaging, and drug/gene/peptide delivery for preventing the active cargo from degradation [14,15,16,17,18,19]. However, previous studies on MSNs for drug delivery application demonstrated that the encapsulation of various therapeutic guest species in the silica frameworks of MSNs is usually dependent on their affinity with the MSN frameworks, which is substantially accomplished through weak interactions between them [20]. In some instances, the loaded drug amount is meager owing to physical adsorption in the mesopores, facilitating their immediate release during encapsulation. To overcome this limitation, our group has synthesized the metal-doped MSNs, in which the metals impregnated in the siliceous frameworks serve as anchors for the drug molecules through establishing the coordination interactions with them [13]. These host–guest interactions between metal and ligand species not only facilitate the higher loading efficiency compared to naked MSNs but also enable their specific delivery in the acidic environment that is prevalent in bacterial infectious sites and tumors [12]. 

Silver has been extensively applied in daily life, and is well known for its antibacterial effect since antiquity [21]. Ultrasmall-sized silver nanoparticles (SNPs) generated through silver-ion reduction have enormous potential towards inhibiting the growth of a broad range of bacteria along with impeding the acquired multidrug resistance (MDR) [22,23,24,25,26]. These positively-charged ultrasmall nanoparticles can attach specifically to the bacterial surface through electrostatic interactions and internalize through damaging the cell wall. Moreover, these internalized tiny reactors generate toxic reactive oxygen species (ROS), which interfere with the metabolic actions of cellular components and lead to the inactivation of bacteria [27,28,29]. However, the bioefficacy of such ultrasmall SNPs significantly depends on various factors, such as their binding efficiency with the surface bioreceptors and the release of silver ions from the SNPs [22]. In this framework, numerous advancements have been further made to enhancing the efficacy of SNP-based antibacterial treatment modalities. In some instances, a combination of antibiotics/drugs with SNPs resulted in the expansion of the antibacterial spectrum [8]. In addition, SNPs are incorporated in the polymer to improve their stability, charge, and substantial antibacterial efficacy [30]. Other approaches include silica coating around the SNP core and decoration with ultrasmall SNPs, or functionalization with pH-responsive silane groups for the release of silver ions [8,17,31,32,33]. Although several studies on antibacterial efficacy have been performed, the combating of antibiotic resistance using SNPs remains inexplicable.

**Figure 1:**
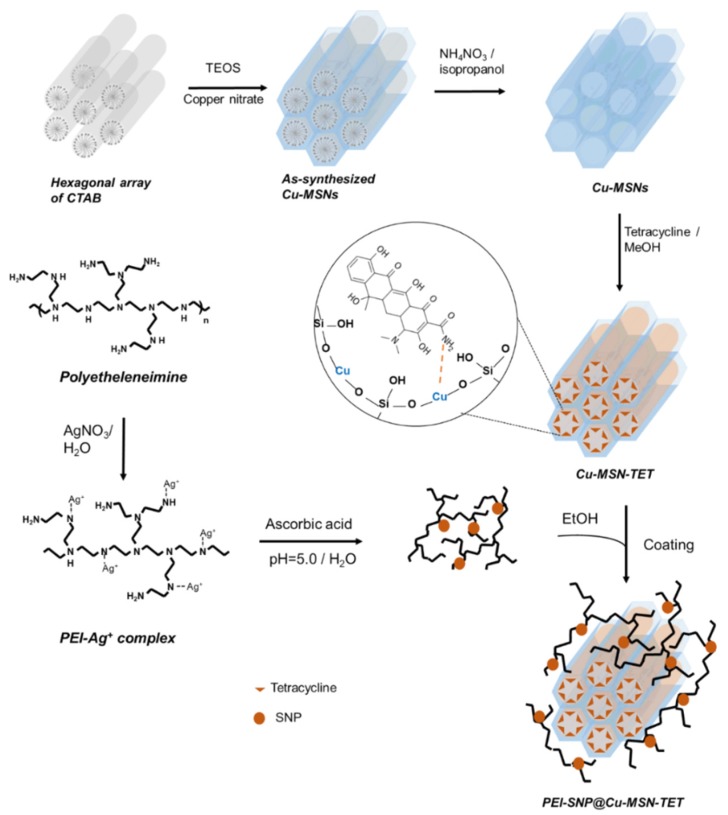
Synthesis outline of the work. Schematic illustrating the outline of the fabrication of the nanohybrids based on Cu-impregnated mesoporous silica nanoparticles (MSNs) and their subsequent coating with the silver nanoparticle (SNP)–deposited polyethyleneimine (PEI) composite layer.

In an attempt to accomplish the fabrication of mesoporous silica-based versatile trio-nanocomposites toward combating antibiotic resistance in bacteria, herein, we designed tetracycline (TET)-immobilized copper-doped MSNs (Cu-MSNs) and further coated with SNP-stabilized polyethyleneimine (PEI-SNP) through a series of synthetic steps (Figure 1). The surface-coated PEI-SNP composite layer and the Cu species impregnated in the mesoporous siliceous frameworks not only protect the TET molecules’ safely from premature release in the physiological fluids but also enhance their therapeutic efficacy through multiple synergistic mechanisms: damaging the bacterial membrane and facilitating efficient internalization, free radical generation through Fenton-like reaction, and the concomitant precise release of TET in the acidic environment. The fabricated nanocomposites were systematically characterized using diverse techniques. Furthermore, the release investigations in vitro were executed to confirm the pH-responsive discharge of TET species through disassembling the coordination interactions between the Cu and TET species, in the acidic environment of bacterial infection sites specifically. Finally, various in vitro antibacterial evaluation tests were performed systematically using *E. coli* as a bacterium model to explore these facts of performance in combating antibiotic resistance efficiently.

## 2. Materials and Methods 

### 2.1. Reagents and Chemicals

Cetyltrimethylammonium bromide (CTAB), copper nitrate trihydrate (Cu(NO_3_)_2_. 3H_2_O), tetraethyl orthosilicate (TEOS, 98%), sodium phosphate dibasic (Na_2_HPO_4_), tetracycline hydrochloride, ammonium hydroxide (NH_4_OH, 30%), 3-(4,5-dimethylthiazol-2-yl)-2,5-diphenyltetrazolium bromide (MTT), 2′,7′-dichlorofluorescein diacetate (DCFDA), sodium hydroxide, polyethyleneimine (PEI, 25 KDa), and dimethyl sulfoxide (DMSO) were acquired from Sigma Co. Ltd. (St. Louis, MO, USA). Silver nitrate (AgNO_3_) was purchased from Showa Chemical Co. Ltd. (Tokyo, Japan). Microbiological-grade agar was purchased from Focus Bio-Science Ltd. (St Lucia, QLD, Australia). Brain heart infusion (BHI) agar, fetal bovine serum (FBS), and Dulbecco’s modified Eagle’s medium-low glucose (DMEM-LG) were purchased from ThermoFisher Scientific Ltd. (Waltham, MA, USA). 

### 2.2. Fabrication of Cu-MSNs

Uniform-sized Cu-doped MSNs were synthesized using the co-condensation method [34]. Initially, CTAB (0.58 g) was added to NH_4_OH (300 mL, 0.51 M) and stirred at 40 °C. Further, the dilute TEOS (0.2 M) was added and stirred vigorously for 4 h. Then, copper nitrate (Si/Cu -30) and TEOS (1.0 M) were added to the mixture and stirred for an additional 2 h. After aging the solution for 16 h at 40 °C, the resultant nanoparticles were collected. The surfactant CTAB in Cu-MSNs was then removed by the chemical extraction approach (0.3 g of NH_4_NO_3_ in 50 mL of isopropanol at 85 °C for 24 h). 

### 2.3. Immobilization of TET in Cu-MSNs

The Cu species in the MSN framework serve as essential anchors for establishing the coordination interactions with the free –NH_2_ group of TET molecules at a nearly neutral pH (~ 7.0). Despite their solubility in water, the TET molecules precipitate at pH-7.0 and could result in low loading efficiency. To overcome this limitation, we used methanol as a solvent for immobilizing the TET species in Cu-MSNs. Briefly, TET (500 mg) was dissolved in methanol and adjusted the pH value to 7.0. Further, the Cu-MSNs (100 mg) sample was then added to the prepared solution and stirred overnight. Finally, the product was centrifuged and suspended in ethanol for further experiments. The calculated loading efficiency of Cu-MSNs was approximately 19% by correlating the recorded absorbance of the supernatant at 363 nm using an ultraviolet-visible (UV-Vis) spectrophotometer.

### 2.4. Deposition of PEI-SNP Composite Layer over Cu-MSNs

Initially, the PEI-SNP complex layer was prepared by the following method [35]. Briefly, PEI (25 μmol) and AgNO_3_ (25 μmol) were dissolved in 100 mL of dd-H_2_O and stirred for 2 h, resulting in the PEI-Ag^+^ complex. Then, ascorbic acid (300 μmol) was added after adjusting the pH value to 5.0. Later, the mixture was stirred for 48 h to let the silver ions completely reduced to SNPs. Further, the PEI-SNP complex layer was collected by centrifugation (26000 rpm) for 1h. Further, this complex was then added to the precentrifuged Cu-MSN-TET sample and stirred overnight. Finally, the resultant PEI-SNP@Cu-MSN-TET nanoparticles were collected. 

### 2.5. Characterizations

Transmission electron microscope (TEM) images were recorded on a Hitachi H-7100 operating at 100 kV (Hitachi High Technologies Corporation, Tokyo, Japan). Fourier transform infrared spectroscopy (FTIR) spectra at the wavenumber range of 4000–400 cm^−1^ were chronicled on a Bruker Alpha spectrometer (Billerica, MA, USA) using the KBr pellet method. The physical composition determination was observed by thermogravimetric analysis (TGA) curves recorded on TGA Q50 V20 (Universal V4.5A TA Instruments, New Castle, PA, USA), scanned until 800 °C at a rate of 20 °C/min under nitrogen purge (20 mL/min). Particle size distribution and zeta(ζ)-potential values based on dynamic light scattering (DLS) measurements were recorded using Malvern-Zetasizer Nano ZS 90 (ZetaPALS, Malvern, Worcestershire, UK). Electron spin resonance (ESR) spectra were recorded on the EMX spectrometer (Bruker, Billerica, MA, USA) at 77 K. The UV-vis absorbance values were recorded using the Genequant-1300 series spectrophotometer (GE Healthcare Biosciences, Pittsburgh, PA, USA). Fluorescence intensity and optical density at 600 nm (OD_600_) were recorded using EnSpire Multilabel plate reader (Perkin Elmer Inc., Santa Clara, CA, USA).

### 2.6. In Vitro Release

To evaluate the pH-triggered release of TET molecules from the designed Cu-MSN containers, we performed the in vitro release study of TET species using the buffers adjusted to various pH values. An accurately weighed solid nanoparticle sample (5 mg) was suspended in PBS (pH-5.5 and 7.4) to mimic the release behavior in various environments (such as endosomal pH at the infectious bacterial site and physiological fluid, respectively at 37 °C, 150 rpm). Aliquots were removed at regular intervals and analyzed by UV at λ_max_ of 363 nm, and the release percentage of TET was determined periodically at their predetermined corresponding time points. 

### 2.7. Biocompatibility

NIH/3T3 (normal fibroblast) cell line was nourished with 10% FBS-supplemented DMEM-LG medium and cultured in a humidified incubator (37 °C, 5% CO_2_).

The biocompatibility of PEI-SNP@Cu-MSN-TET was measured using the MTT assay [36]. Cells were suspended in 10% FBS-supplemented DMEM-LG medium and seeded in a 96-well plate (1 × 10^4^ cells/well) and incubated for 24 h. Then, the nanoparticles suspended in the FBS-free DMEM-LG at the required concentrations, along with control, were added. After 4 h, FBS containing DMEM-LG medium was added to each well for providing nutrients, and then incubated for another 20 h. Further, the wells were washed twice with lukewarm PBS. Further, 50 μL of MTT solution (1 mg/mL) was added and incubated for another 4 h. Finally, DMSO (150 μL) was added to dissolve the violet crystals (formazan), and the reduced MTT was recorded at 570 nm. The viability was calculated by relatively corroborating the readings with the blank control group.

### 2.8. Antibacterial Efficacy

#### 2.8.1. Bacteria Culture

Prior to the experiment, the preserved *E. coli* strain was sub-cultured in Luria–Bertani broth (LB broth) and incubated at 37 °C overnight. Further, the culture was streaked onto a 100 mm LB-agar plate (quadrant-streaking), and one of the signal colonies was picked up and incubated by providing the same conditions during further experiments [24]. The rationale behind choosing the *E. coli* strain as a testing model is that the PEI polymer is an effective permeabilizer of gram negative strain [37].

#### 2.8.2. Bacteria Viability

Initially, the nanoparticles were washed once each with EtOH and LB broth and were suspended in LB broth containing the *E. coli* bacterial strain at the required concentrations for 24 h. To determine the exact viability of bacteria, the OD_600_ values of the similar dilutions of nanoparticles and bacteria were read distinctly as blank and control for removing the sample interference. The OD_600_ value of the bacteria was measured and calculated by the following Equation (1):

(1) $$A=A_{x}-A_{blank}$$

where *A_x_* and *A_blank_* are OD_600_ values at certain time intervals and respective nanoparticle samples alone. The EC_50_ is the concentration where *A*/*A_control_* at 50%.

Further, the minimum inhibitory concentration (MIC_50_) was recorded with a similar protocol, but the density of bacteria was measured by the following procedure. After treatment with various concentrations of different nanoparticle samples and at altered periods of incubation, the bacterial broth was diluted 10^7^ times, and 10 µL of diluted bacterial broth was spread on the LB-agar plate. The bacterial viability curves were obtained from counting the colony-forming units (CFU) number on the LB-agar plate and calculated by the following Equation (2):

(2) $$\mathrm{Cell viability}(\%)=\frac{{\mathrm{CFU}}_{x}}{{\mathrm{CFU}}_{0}}\times 100$$

where CFU_x_ is the CFU number from the group treated with nanoparticles, CFU_0_ is the CFU number from the control group. MIC_50_ is the concentration, where the bacterial viability reaches 50%.

#### 2.8.3. Bacteria Growth Study

The bacterial broth was diluted to 8 × 10^7^ CFU/mL and incubated with a serial dilution of nanoparticles. The density of bacteria was then measured by recording the OD_600_ at the specific time intervals (1st, 2nd, 3rd, 4th, 7th, 12th, and 24th h) and calculated using the Equation (2).

#### 2.8.4. CFU Assay

The MDR *E. coli* strain was grown in LB broth, and 100 μL of the broth was inoculated in the fresh culture medium prior to the experiment to resume the bacteria cell cycle. After 1 h of incubation, the bacteria were synchronized, and the viable cells were optimized by fixing the OD_600_ as 0.2. Subsequently, the modicum aliquots (10 µL) approximately possessing 1.6 × 10^8^ CFU/mL were incubated with various nanoparticles and incubated at 37 °C. Finally, the aliquots were diluted and spread on an agar plate and incubated overnight [38]. 

#### 2.8.5. Determination of ROS 

The ROS levels in the bacteria were determined using the DCFDA assay [39]. When the bacterial membrane was being damaged by the nanocomposites, the generated cellular ROS further rapidly oxidized the fluorogenic dye, DCFDA to highly fluorescent DCF. For the treatment groups, the cells were diluted with LB broth to 1.6 × 10^7^ CFU/mL (OD_600_ is about 0.02). Further, the bacteria were treated with nanoparticles (200 μg/mL) and incubated for 6 h. Then, the supernatant was separated by centrifugation and transferred into a 96-well microplate. Next, the DCFDA solution was added into each well (final concentration of 100 μM) and incubated for 30 min. Finally, the fluorescence of DCF was detected by a fluorescence plate reader at *E_x_*/*E_m_* = 485/535 nm.

#### 2.8.6. Membrane Permeability Assay

To determine the bacterial membrane permeability, herein, we used propidium iodide (PI) staining assay following the reported procedure [40,41]. Briefly, the bacterial broth was initially diluted to 8 × 10^7^ CFU/mL and treated with the designed nanocomposites (20 µg/mL), in addition to the negative as well as positive control groups, of media treatment and 70% isopropanol, respectively. After 6 h of incubation, the PI stain (20 µg/mL) was added to the bacterial suspension and incubated for a further 15 min. The bacteria culture was washed twice and suspended in PBS. Finally, the fluorescence of PI was measured (λ_Ex/Em_ -536/623 nm) within 2 h using the flow cytometer (BD Accuri-C5).

### 2.9. Statistical Analysis

All data were expressed as mean ± standard deviations (SD, *n* = 3) and compared using analysis of variance (ANOVA) using Tukey’s honestly significant difference at a significance of *p* < 0.05. 

## 3. Results and Discussion

In this study, we fabricated innovative mesoporous silica-based trio-nanohybrids using a multi-step approach for combating antibiotic resistance in bacteria. To improve the loading efficiency of the antibiotic herein, TET, we initially doped the Cu species in the siliceous frameworks for effectively anchoring drugs with the amine functional group through the host–guest chemistry-assisted coordination interactions. In addition to augmented loading efficiency, these doped Cu species in the silica framework trigger the release of TET species precisely in the acidic environment at the infection sites. In addition, the impregnated Cu species synergizes the antibacterial efficacy of immobilized TET species by generating Fenton-like reaction-catalyzed toxic free radicals. Indeed, the TET molecules inhibit bacterial growth through various mechanisms, such as binding to ribosomes and concomitant protein synthesis inhibition. Further, the silver ions reduced to SNPs are instantaneously dispersed in the PEI polymer and eventually coated over the Cu-MSN surface for enhancing the antimicrobial activity silver and surpassing MDR. To explore these facts, we have systematically characterized this hierarchical design and further elucidated the antibacterial efficacy.

### 3.1. Characterizations

**Figure 2:**
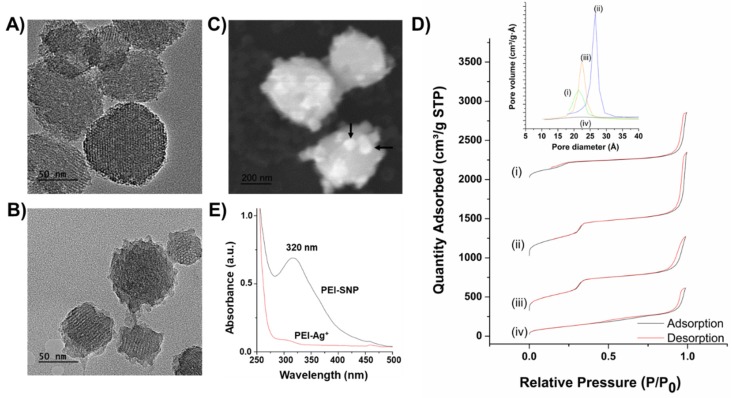
Characterizations of the designed nanocomposites. TEM images of (**A**) Cu-MSNs and (**B**) PEI-SNP@Cu-MSNs (scale bar: 50 nm). (**C**) SEM image of PEI-SNP@Cu-MSNs (scale bar: 200 nm). (**D**) Brunauer–Emmett–Teller (BET) curves of the respective modified samples: (i) pristine Cu-MSNs, (ii) surfactant template-extracted Cu-MSNs, (iii) Cu-MSN-tetracycline (TET), and (iv) PEI-SNP@Cu-MSN-TET. (**E**) UV-vis spectra of PEI-SNP composite layer illustrating the formation of SNPs, i.e., before (red) and after (black) SNP formation.

As illustrated in Figure 2A,B, the surface morphology of synthesized Cu-MSNs, as well as PEI-SNP complex-coated Cu-MSN composites, was demonstrated using the TEM observations. The surfactant-extracted Cu-MSNs were uniformly distributed with an average size of around 150 nm. After coating with the PEI-SNP complex, the surface of the nanocomposites was a bit dappled due to the distribution of small-sized SNPs in the PEI matrix (Figure 2B). However, the distribution of the SNPs could be well-observed from the SEM images (white dots directed by black-colored arrows, Figure 2C), indicating that the low contrast casing around the Cu-MSNs signifying the successful coating of PEI-SNP layer around Cu-MSNs. Further, the textural properties of MSNs elucidating the surface area, and other structural parameters were chronicled using Brunauer–Emmett–Teller (BET) theory. The adsorption isotherm curves represented the type-IV gas adsorption pattern with a small hysteresis loop based on International Union of Pure and Applied Chemistry (IUPAC) classification. The adsorption isotherms and textural properties are presented in Figure 2D and Table 1, respectively. The as-synthesized Cu-MSNs yielded the low surface area of 436 m^2^/g (Figure 2D-i), which subsequently increased to 1317 m^2^/g after surfactant extraction, attributing to the high degree of extraction of CTAB in NH_4_NO_3_/isopropanol mixture (Figure 2D-ii). Further, the immobilization of TET ensued in a slight reduction in the eventual surface area of Cu-MSNs due to their occupancy in the pores (Figure 2D-iii). Subsequently, the BET surface area was significantly reduced to 445 m^2^/g after coating with the PEI-SNP layer, indicating that the SNP-stabilized polymeric matrix was shielded over the mesopores (Figure 2D-iv). Further, the characteristic mesostructured properties elucidating the pore volume, as well as the diameter of Cu-MSNs, also followed a similar trend with the surface area, as summarized in Table 1. 

From the UV-vis measurements, a single broad peak centered at around 320 nm owing to the Surface Plasmon Resonance (SPR) characteristics of silver was observed, attributed to the successful silver ion reduction to the SNPs (Figure 2E). Moreover, as anticipated, it could be concluded that the resultant SNPs were no larger than 40 nm, indicating no signs of aggregation. These experimental results were in accordance with the TEM observations [42]. Further, the zeta potential values are also in agreement with the results, stating that the Cu-MSN sample after PEI-SNP coating resulted in a higher positive charge (+32.0 mV) compared to its preceding samples, which could efficiently increase the affinity of the nanoconjugates toward the negatively-charged bacterial cell surface. Notably, the surface charge of the Cu-doped MSNs (Cu-MSN-ext) resulted in a higher positive charge than conventional MSNs, indicating the successful encapsulation of Cu species in MSNs. These doped Cu species in MSNs significantly augment the loading efficiency through establishing the coordination linkage in addition to weak hydrogen bonding patterns between the host and guest molecules (Si–O^−^⋯HN-TET). As a result, the TET-loaded Cu-MSNs sample resulted in the reduced zeta potential value and shifted toward the negative trend, confirming the successful encapsulation of TET species in the mesopores.

**Figure 3:**
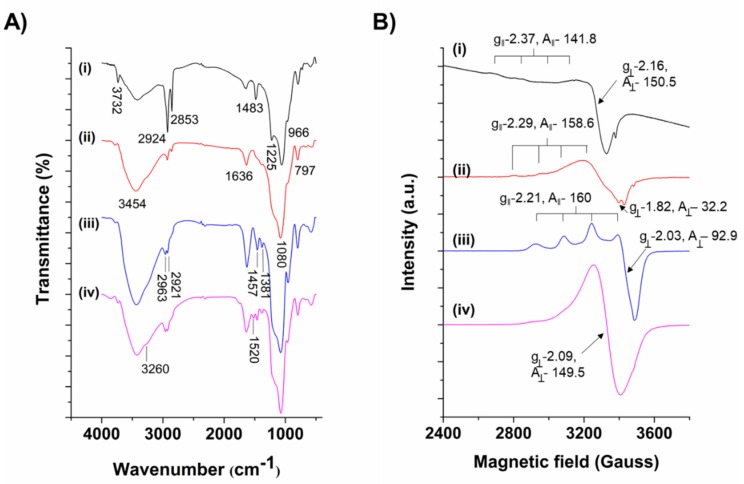
Characterization of the designed nanocomposites. (**A**) Fourier-transform infrared spectroscopy (FTIR) spectra exploring various chemical functionalities of Cu-MSNs and their successive samples, (i) pristine Cu-MSNs, (ii) surfactant template-extracted Cu-MSNs, (iii) Cu-MSN-TET, and (iv) PEI-SNP@Cu-MSN-TET. (**B**) Electron spin resonance (ESR) spectra of (i) Cu-MSNs, (ii) Cu-MSN-TET, (iii) PEI-SNP@Cu-MSN-TET, and (iv) the spectrum of PEI-SNP@Cu-MSN-TET, recorded after the release study in the buffered saline (pH-5.5).

The chemical functionalities of Cu-MSNs and their successive modifications were chronicled using the FTIR analysis (Figure 3A). A strong and broad peak centered at around 3454 cm^−1^ could be ascribed to the O–H stretching vibrations, and a relatively weaker peak at 1636 cm^−1^ could be attributed to the bending vibrations of the absorbed water molecules in all the samples. The peaks at 3732, 2924, and 2853, as well as 1483 cm^−1^, could be attributed to quaternary ammonium cation, C–H stretching, and C–H bending vibrations of the CTAB molecules in the pristine Cu-MSNs, respectively (Figure 3A-i). After surfactant extraction, the prominent peaks described above were significantly reduced, confirming the CTAB removal and generation of mesopores (Figure 3A-ii). In Cu-MSNs, the peaks at around 1080 and 797 cm^−1^ could be assigned to the vibrations of characteristic silanol vibrations of the siliceous frameworks [43]. The peaks at 2963 and 2921 cm^−1^ could be attributed to the C–H stretching, and other C–H vibrations, respectively. A peak at 1457 cm^−1^ due to the aromatic C–H bending and a weak peak at around 1381 cm^−1^ could be attributed to the C–H bending of the methyl group (–CH_3_), indicating that the TET molecules were successfully immobilized in the Cu-MSNs (Figure 3A-iii) [44]. After coating with the PEI-SNP composite layer, the Cu-MSNs resulted in additional peaks at 1520 and 3260 cm^−1^, which could be attributed to the N–H bending and N–H stretch of PEI, respectively, endorsing the successful deposition of the polymeric matrix over Cu-MSNs (Figure 3A-iv).

Further, the ESR analysis was performed to explore the structural paramagnetic characteristics of Cu species impregnation in the siliceous frameworks. In addition, the respective g and A values of the ESR spectra were enumerated after successive modifications (Figure 3B). The Cu-MSNs sample exhibited an apparent hyperfine splitting of Cu(II) with g_||_ at 2.37, and g_⊥_ at 2.16, indicating that the metal species were effectively synchronized in the mesoporous siliceous network with a distorted square pyramidal octahedral conformation (Figure 3B-i) [45]. However, after loading the TET molecules, some changes were observed such as a shift in g_e_ to 1.82 and the clear splitting at the higher magnetic field (~3400 G), indicating that the drug molecules were successfully conjugated to the Cu species in the framework through the coordination interactions (Figure 3B-ii). Further, the PEI-SNP@Cu-MSN-TET sample exhibited hyperfine splitting of Cu(II) with a nuclear spin I=3/2 in all the g values (g_||_ at 2.21 and g_⊥_ at 2.03) (Figure 3B-iii). In addition to the Cu doping and the loading efficiency of TET in MSNs, we further explored the pH-responsive disassembly nature of the established coordination linkage between TET and MSNs species by dispersing the nanocarriers in the buffer solution (pH-5.5). It was observed that the disappearance of hyperfine spitting from the PEI effect, similar to the spectra of Cu-MSNs, was regained with a relatively similar spectrum shape (Figure 3B-iv). Together, these experimental results indicated that the Cu species were successfully doped in the siliceous frameworks, and subsequent incorporation of TET species, as well as their encapsulation in the PEI-SNP matrix, were also evident, which could potentially allow for the use of such innovative pH-responsive nanocomposites for drug delivery to the infection sites precisely.

Further, the loading efficiency of TET molecules in Cu-MSNs and their thermal stability were demonstrated by enumerating the successive weight loss events of various nanocarriers using the TGA analysis (Figure S1). The Cu-MSNs sample resulted in no significant weight loss events (Figure S1-a) except a slight weight loss (~5%) before 100 °C due to the atmospheric moisture, and the surface adsorbed water molecules in the sample. However, a significant weight-loss event (~18 Wt.%) in the TET-loaded Cu-MSNs sample was observed after 400 °C, which could be attributed to the loss of the immobilized drug molecules (Figure S1-b). The loading efficiency was in accordance with the UV-vis recordings (see Section 2.3). After coating with the PEI-SNP-based composite layer, a series of degradation events were observed after 185 °C, attributing to the highly sensitive PEI degradation coated over the surface of Cu-MSNs. However, the weight loss was lesser compared to the prior sample due to the extensive distribution of stable SNPs in the PEI matrix (Figure S1-c). Furthermore, we also observed a shift in the degradation temperature of TET, demonstrating that the nanosized mesopores of Cu-MSNs provided a stable gallery for the encapsulated drug molecules. 

### 3.2. In vitro TET Release 

**Figure 4:**
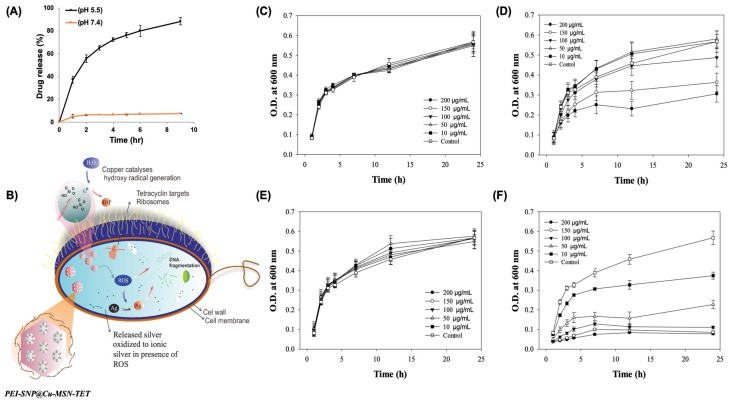
In vitro drug release and antibacterial efficacy of the designed nanocomposites. (**A**) TET release from Cu-MSNs at various time intervals in simulated physiological fluids (phosphate-buffered saline (PBS) at pH values of 5.5 and 7.4). (**B**) Schematic illustrating the plausible mechanism of the designed PEI-SNP@Cu-MSN-TET nanocomposites after their successful internalization. Effect of Cu-MSNs and their successive nanoconjugates on the growth of resistant *E. coli* strain at different concentrations of (**C**) Cu-MSNs, (**D**) Cu-MSN-TET, (**E**) PEI-SNP@Cu-MSNs, and (**F**) PEI-SNP@Cu-MSN-TET.

In addition to the augmented loading efficiency, the installed Cu metal species in the MSNs facilitate the triggered release of drugs in the acidic microenvironment. The discharge behavior of encapsulated guest TET species was demonstrated in various buffers that mimicked the pH values of the infectious bacterial site (pH-5.5) and blood (pH-7.4) (Figure 4A). As anticipated, the release of TET in vitro at pH-5.5 was significantly higher compared to that of the buffer at pH-7.4, demonstrating the sensitivity of the coordination linkage between the host and guest species, TET and Cu, precisely in the endosomal environment. It should be noted that this feature of a responsive release, specifically in the infectious site, could efficiently deliver the therapeutic cargo without any premature release in the bloodstream.

### 3.3. Biocompatibility

Although numerous studies have shown that the MSNs are considerably biocompatible both in vitro and in vivo, the utilization of different components during successive modifications would lead to severe compatibility issues, which stringently demand biosafety assessment of the designed nanocomposites. To explore the biocompatibility of PEI-SNP@Cu-MSN-TET composites, the cytotoxicity in vitro of the nanoparticles was performed using MTT assay in normal fibroblast cells (3T3 cells). The cells were subjected to PEI-SNP@Cu-MSN-TET sample treatment at various concentrations (0-200 µg/mL) for 24 h. The experimental results showed greater than 90% cell viabilities at all the concentrations of nanocomposites, indicating that the designed nanocarriers resulted in no any significant effects, attributing to the high biocompatibility of siliceous frameworks and polymeric matrix as well as the excellent suspension ability of these fabricated nanocomposites (Figure S2).

### 3.4. Antibacterial Efficacy

Further, the antibacterial efficacy of these designed nanocomposites was determined through various tests, such as the CFU assay using nonresistant as well as resistant strains of bacteria, determination of ROS levels using DCFDA method, and PI staining by flow cytometry for the extent of bacterial membrane damage. Prior to the efficacy assessment, the plausible mechanism of the designed trio-nanocomposites toward complete devastation of bacterial strains by overcoming MDR is anticipated as follows. Initially, the positively-charged SNPs deposited over the Cu-MSN surface establish the intimacy with the negatively-charged bacterial surface. Further, the decorated positively-charged SNPs substantially attach to the bacterial cell wall and damage it through releasing the silver ions, which substantially enhance the permeation of the nanoconstructs and finally exchange the cellular components with the extracellular fluids resulting in an imbalance of the infectious environment (Figure 4B). Subsequently, the internalized nanoparticles release the TET molecules intracellularly through disassembling the coordination interactions as they are highly susceptible and trigger its release in an acidic environment since the infectious site is acidic due to the secretion of organic acids and the activation of the natural immune system under hypoxic conditions. Intracellularly released TET molecules inactivate ribosomes and subsequently inhibits protein synthesis. The synergistic antibacterial effectiveness of MSN trio-hybrids using different metals (in this case, Cu and Ag) and antibiotics (TET) is favorable through multiple mechanisms. In a way, the Cu species in the mesoporous framework facilitate the free radical generation, i.e., ROS through the Fenton-like reaction intracellularly, in addition to silver ion release from SNPs. These intrinsic ROS levels and their insults cause substantial lethal damage to the bacteria. Moreover, the silver ions improve the access of TET to cell components by favoring the bacterial membrane damage. Together, these slackening consequences weaken the ability to recover the cell wall integrity and ablate bacteria through mentioned multiple actions by overcoming the antibiotic resistance.

Initially, the bacterial growth was enumerated after treatment with various concentrations of the designed nanocomposites. Further, the viability of bacteria in the presence of respective TET-molecule-encapsulated Cu-MSN composites in the *E. coli* strains resulted in both time-dependent (Figure 4 and Figure S3) and dose-dependent (Figures S4 and S5) inhibition effects. The antibacterial efficacy of TET molecules could be due to their duly release facilitated by the slackening of the coordination interactions, specifically in the acidic environment. As anticipated, the inhibition rate of the Cu-MSN-TET sample was higher in the nonresistant strain (Figure S3-B) compared to resistant strain (Figure 4-D), with only 54% of survival rate at a concentration of 200 μg/mL. However, the Cu-MSN-TET nanoconjugates were highly capable of inhibiting the bacterial growth at the anticipated levels beyond 150 μg/mL, due to the synergistic effects offered by Cu, but could not substantially arrest the bacterial growth. Further, the inhibition rate of PEI-SNP-coated Cu-MSN composites was comparatively higher than that of their preceding sample, Cu-MSN-TET in both the strains and at least, i.e., 14% of the equivalent dose of PEI-SNP@Cu-MSN-TET in the resistant strain, which could be attributed to the efficacy of dispersed SNPs in the PEI matrix (Figure 4F). The obtained MIC value of PEI-SNP@Cu-MSN-TET was around 21.1 μg/mL (Figure S4). Moreover, with the increasing time of exposure (1^st^, 2^nd^, 3^rd^, 4^th^, 7^th^, 12^th^, and 24^th^ h), the synthesized nanoconstructs resulted in efficient bacterial growth inhibition in both the resistant and nonresistant bacterial strains. Remarkably, with the increase of concentrations (0–200 µg/mL), Cu-MSNs and PEI-SNP@Cu-MSNs resulted in no effect even at higher doses in both the resistant and nonresistant strains, respectively, attributing to the biocompatibility of the MSN frameworks and polymeric matrix (Figure S5). Although the silver species in PEI-SNP@Cu-MSNs would damage the cell wall, which, however, could recover after damage. 

**Figure 5:**
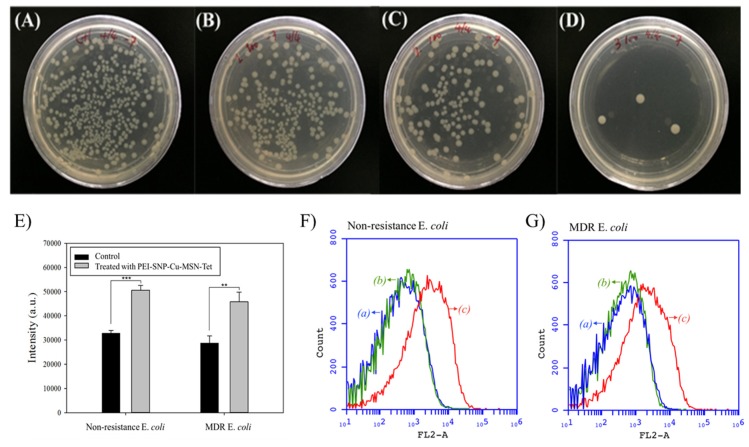
Images showing a variable number of colony-forming units (CFUs) of drug-resistant strain of *E. coli* after supplementation with Cu-MSNs along with its successive conjugates at a concentration of 100 μg/mL: (**A**) Control, (**B**) Cu-MSNs, (**C**) Cu-MSN-TET, and (**D**) PEI-SNP@Cu-MSN-TET. (**E**) ROS levels in both nonresistance and multidrug-resistance (MDR) *E. coli* strains treated with the PEI-SNP@Cu-MSN-TET sample at 200 μg/mL. ** *p* < 0.01 and *** *p* < 0.001. Bacterial membrane permeability of (**F**) nonresistant and (**G**) resistant *E. coli* strains by flow cytometry measurements of propidium iodide (PI) stain in various treatments, (a) control, (b) Cu-MSN-TET, and (c) PEI-SNP@Cu-MSN-TET.

Despite the resistance gained by bacteria toward the drug, the negatively-charged bacterial cell membrane renders the intimacy with the positively-charged silver species on its surface, facilitating their efficient internalization and substantial delivery of the drug cargo in the acidic microenvironment. Further, the antibacterial efficiency of the fabricated MSN-based composites was qualitatively measured by enumerating the CFUs of the resistant *E. coli* strain after incubation with nanoparticle suspensions (100 μg/mL) for 24 h (Figure 5A–D). The assay was further continued by diluting the modicum bacterial solution to 10^7^ times and spread on predried Luria–Bertani (LB)-agar media plates. The TET molecules-loaded samples, i.e., Cu-MSN-TET, and PEI-SNP@Cu-MSN-TET, resulted in fewer colonies of resistant bacterial strain compared to control and bare Cu-MSNs, indicating the efficient release of drug intracellularly. At the outset, the CFUs were significantly lesser in the PEI-SNP@Cu-MSN-TET sample with just three in number compared to other samples, attributed to the synergistic effects of MSN-based trio-constructs, i.e., TET, Cu, and SNPs. More often, the silver ions from the SNPs generate immense ROS levels, which interfere with the metabolic activities of the cellular components. In addition, the Cu species in the mesoporous frameworks generate ROS through hydrogen peroxide-catalyzed Fenton-like reaction. To explore the generation of lethal ROS levels intracellularly, we measured the free radical species using a cell-permeant reagent DCFDA based cellular detection assay. Various free radical species intracellularly were measured by correlating the oxidation of nonfluorescent DCFDA to a highly fluorescent molecule, DCF, by ROS. The ROS levels were considerably higher in bacteria treated with the PEI-SNP@Cu-MSN-TET composites over the control treatments (media alone) in both the nonresistant and resistant strains of *E. coli* (Figure 5E). These free radical species from SNPs and Cu species in the framework could damage the bacterial membrane, which might be the plausible reason for the antibacterial efficacy of the designed nanoconjugates. 

Indeed, the excess intracellular ROS levels outburst the bacteria, leading to the expulsion of the cellular components. In addition, these slackening consequences also result in the exchange of extracellular fluids due to high permeability. To explore the bacterial membrane permeability, the PI stain is highly convenient, as it permeates explicitly through the damaged/injured cell membrane. Intracellular PI levels were quantified using flow cytometry after treatment with various nanoconjugates, correlating to the extent of bacterial membrane damage. As illustrated in Figure 5F, G, the PEI-SNP@Cu-MSN-TET sample treated bacterial membrane was more susceptible to the permeability of the PI stain compared to other treatments of control and Cu-MSN-TET sample in both nonresistant and resistant strains of bacteria. However, a very similar trend was observed in the treatment groups of both the bacterial strains. Nevertheless, the treatment of PEI-SNP-coated Cu-MSNs sample showed that cells possessed higher accumulation of PI fluorescence compared to the bare nanocomposites.

## 4. Conclusions

In summary, we demonstrated the design of well-ordered, biocompatible Cu-doped MSNs with an extensive specific surface area, subsequently encapsulated with TET species, and coated with PEI-SNP complex layer to combat antibiotic resistance in bacteria. The loading capacity of TET in the designed nanoparticles was very high and quite sufficient to achieve the availability demand in bacteria due to MDR. The designed nanoconjugates were systematically characterized, and the results were highly commendable. The bioassay results indicated that SNPs in the design significantly improved the antibacterial ability in MDR *E. coli* strain through sensitizing the cell membrane and improved the intracellular availability of nanocontainers for pH-triggered delivery of drug cargo. In addition, enormous ROS levels due to Cu species in the framework facilitated the devastation of MDR bacteria. This trio-constructs-based nanoparticle design is a promising approach and could be utilized for fabricating the drug delivery systems to treat various bacterial infections clinically.

## Figures and Tables

**Table 1:** BET method-assisted textural properties and dynamic light scattering (DLS)-based zeta potential values of various samples of Cu-MSNs.

Sample	BET Surface Area (m²/g)	Pore Volume (cm³/g)	Pore Size (nm)	Particle Size (nm)	Zeta potential (mV)
Pristine Cu-MSNs	436	0.81	2.18	140.5	32.8 ± 1.3
Cu-MSN-ext	1317	1.89	2.76	187.1	−17.5 ± 1.1
Cu-MSN-TET	1133	1.65	2.26	246.5	−30.8 ± 1.5
PEI-SNP@Cu-MSN-TET	445	0.95		256.8	32.0 ± 0.7

## Supplementary Materials

The following are available online at https://www.mdpi.com/2079-4991/10/3/597/s1, Figure S1: TGA curves, Figure S2: Biocompatibility study, Figure S3: Bacterial growth studies of nonresistance *E. coli* strain, Figure S4: MIC calculations, and Figure S5: Antibacterial efficacy.
