# Supplementary material for: Combating Antibiotic Resistance through the Synergistic Effects of Mesoporous Silica-Based Hierarchical Nanocomposites
Source: Nanomaterials (Basel). 2020 Mar 24;10(3):597. doi: 10.3390/nano10030597 (PMC7153717; doi:10.3390/nano10030597)
Supplement: Supplementary file 1 [file nanomaterials-10-00597-s001.pdf]

Supplementary Materials

Combating Antibiotic Resistance Through the Synergistic Effects of Mesoporous Silica-Based Hierarchical Nanocomposites

Ranjith Kumar Kankala[1,2,†], Wei-Zhi Lin[1,†] and Chia-Hung Lee[1,*]

[1] Department of Life Science, National Dong Hwa University, Hualien 97401, Taiwan; ranjithkankala@hqu.edu.cn (R.K.K.), ex20122@gmail.com (W.-Z.L.)
[2] College of Chemical Engineering, Huaqiao University; Xiamen 361021, China
* Correspondence: chlee016@gms.ndhu.edu.tw; Tel.: +886-3-890-3677
† These authors contributed equally to this work.

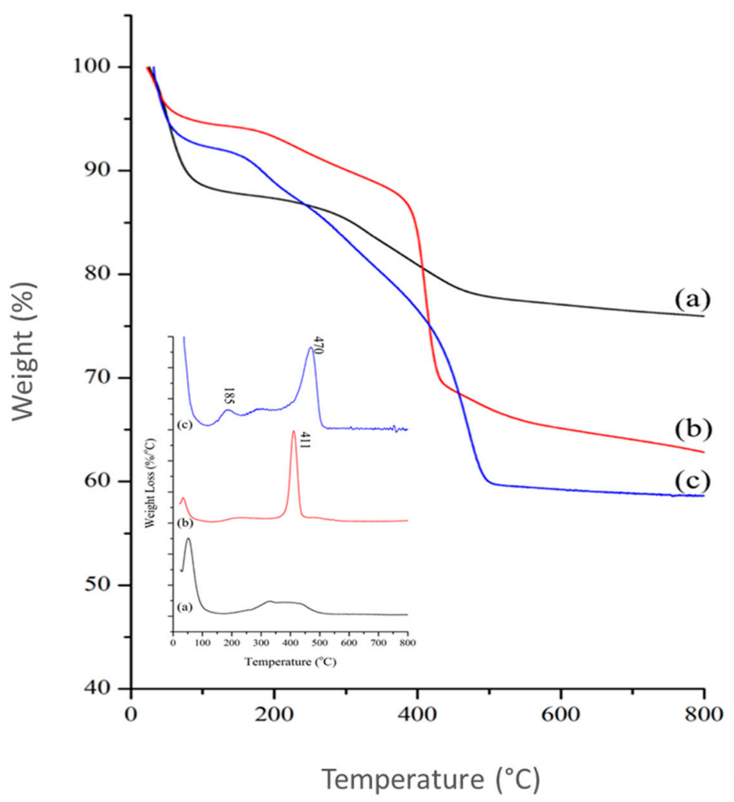

Figure S1. TGA curves of (a) Cu-MSN, (b) Cu-MSN-TET, and (c) PEI-SNP@Cu-MSN-TET. Inset showing the corresponding differential curves.

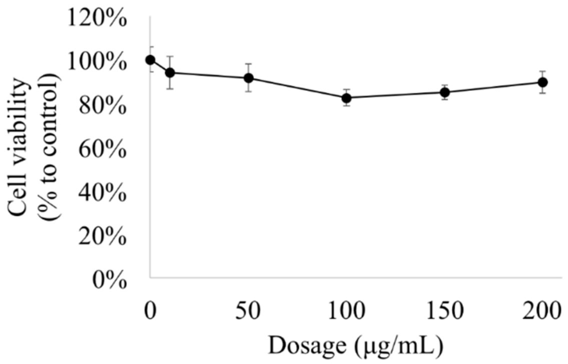

Figure S2. Biocompatibility study of PEI-SNP@Cu-MSN-TET nanoconjugates using MTT cytotoxicity assay in normal fibroblast cell line (3T3 cells).

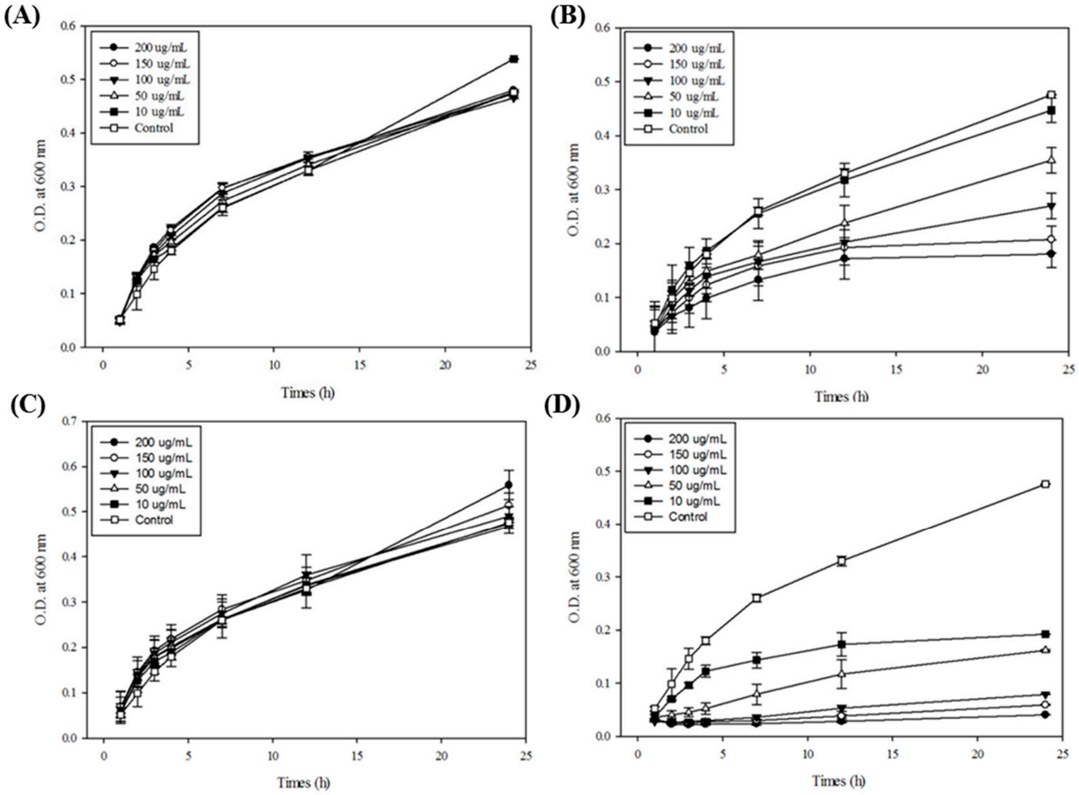

Figure S3. The bacterial growth studies of non-resistance E. coli strain. The growth curves from bacteria treated with (a) Cu-MSN, (b) Cu-MSN-TET, (c) PEI-SNP@Cu-MSN, (d) PEI-SNP@Cu-MSN-TET.

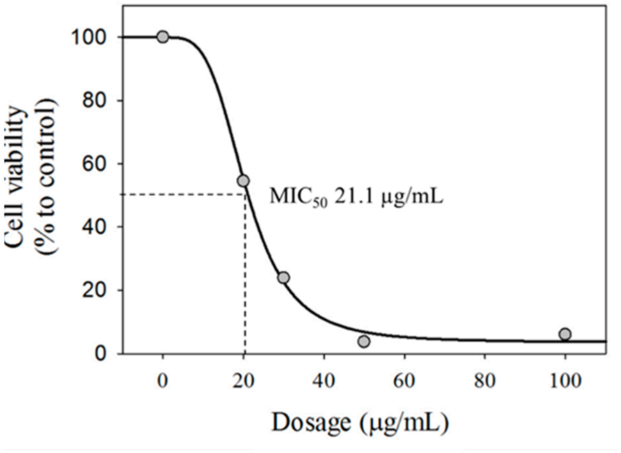

Figure S4. MIC calculation of PEI-SNP@Cu-MSN-TET sample in MDR E. coli strain. Bacteria was treated with a serial dosage of nanoparticles and incubated for 24 h.

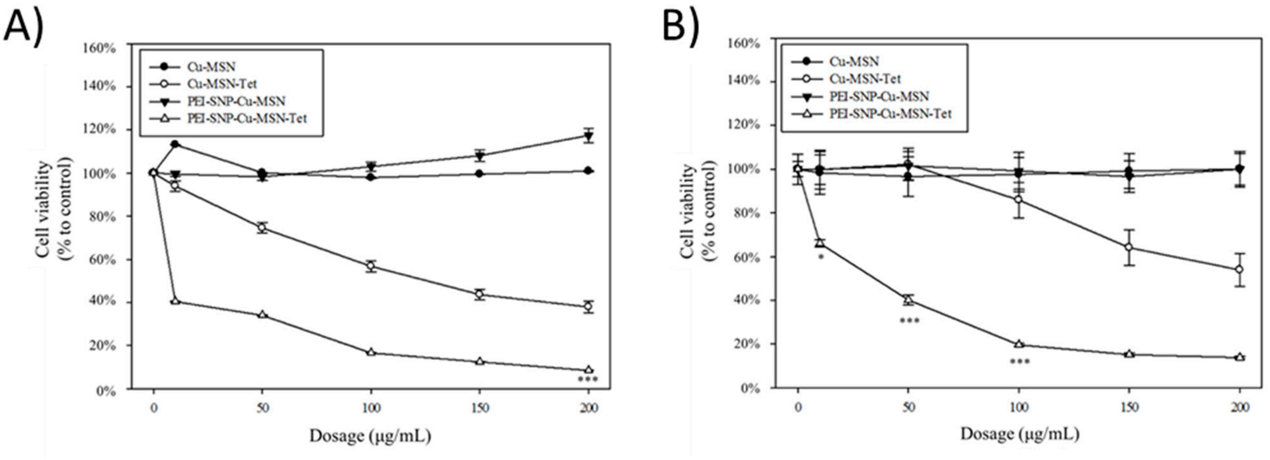

Figure S5. The antibacterial activity of nanoparticles on (A) non-resistance E. coli strain and (B) MDR E. coli strain. Bacteria was treated with a serial dosage of nanoparticles and incubated for 24 h. *p value < 0.05, and *** p value < 0.001 with control.
